# Comparative Analysis of Bacterial Diversity and Functional Potential in Two Athalassohaline Lagoons in the Monegros Desert (NE Spain)

**DOI:** 10.3390/microorganisms13102224

**Published:** 2025-09-23

**Authors:** Mercedes Berlanga, Arnau Blasco, Ricardo Guerrero, Andrea Butturini, Jordi Urmeneta

**Affiliations:** 1 Departament de Biologia, Sanitat i Mediambient, Secció de Microbiologia, Facultat de Farmàcia i Ciències de l’Alimentació, Universitat de Barcelona, 08028 Barcelona, Spain; 2 Departament de Biologia Evolutiva, Ecologia i Ciencies Ambientals, Facultat de Biologia, Universitat de Barcelona, 08028 Barcelona, Spain; 3 Laboratori de Microbiologia Molecular i Antimicrobians, Departament de Patologia i Experimental Terapèutica, Facultat de Medicina, Universitat de Barcelona, 08907 Hospitalet del Llobregat, Spain; 4 Departament de Genètica, Microbiología i Estadística, Facultat de Biologia, Universitat de Barcelona, 08028 Barcelona, Spain

**Keywords:** Monegros desert, endorheic saline lagoons, Salineta and La Muerte lagoons, bacterial community diversity

## Abstract

This study compared bacterial diversity and putative functionality between two saline lagoons, La Muerte and Salineta, in the Monegros desert ecosystem. Amplicon sequencing analysis revealed distinct taxonomic and functional patterns between the lagoons. Pseudomonadota dominated both systems, averaging 31.0% in La Muerte and 47.4% in Salineta, reflecting their well-documented osmotic stress tolerance. However, significant compositional differences were observed: Cyanobacteriota comprised 18.4% of La Muerte communities but remained below 1% in Salineta, while Bacteroidota showed higher abundance in La Muerte (16.6%) compared to Salineta (6.7%). Principal coordinate analysis demonstrated strong community differentiation between lagoons (Bray–Curtis dissimilarity *p* < 0.05). Functional profiling revealed contrasting metabolic capabilities: La Muerte communities showed enhanced autotrophic carbon fixation pathways (especially the Calvin–Benson cycle) and nitrogen cycling processes, while Salineta exhibited stronger denitrification signatures indicative of anoxic conditions. Carbohydrate indices suggested different organic matter quality and polymer composition between lagoons. La Muerte demonstrated significantly elevated stress response mechanisms compared to Salineta, which can be attributed to its ephemeral, shallow, and high evaporation rates that collectively generate more severe osmotic, thermal, and oxidative stress conditions for the sediment microbiota. These findings demonstrate that site-specific environmental factors, including hydroperiod variability and salinity dynamics, strongly influence microbial community structure and metabolic potential in saline wetland ecosystems.

## 1. Introduction

Endorheic basins—closed drainage systems in which water leaves the catchment only by evaporation or infiltration—occupy almost 10% of the Earth’s continental area and constitute some of the planet’s most dynamic aquatic ecosystems [1]. Because the incoming waters have no surface outflow, evaporation concentrates the dissolved load, and many basins evolve into hypersaline lakes or wetlands that simultaneously experience large daily temperature amplitudes, intense UV irradiation, and nutrient scarcity [2]. These “poly-extreme” habitats are globally widespread in arid belts on every continent, yet they are distinctly uncommon in Europe; the semi-arid Ebro depression in north-eastern Spain hosts the only large complex of inland, shallow athalassohaline lagoons on the continent [3,4]. Despite their scarcity, European endorheic wetlands deliver valuable ecosystem services, act as key stop-over sites for Palearctic migratory birds, and are therefore protected under international frameworks such as the EU Habitats Directive (https://environment.ec.europa.eu/topics/nature-and-biodiversity/habitats-directive_en, accessed on 19 September 2025).

Monegros lagoons lie on Miocene gypsum and marl deposits of the central Ebro Basin. Seasonal precipitation (≲350 mm yr^−1^) and high evapotranspiration (>800 mm yr^−1^) create a strongly negative water balance, meaning that most lagoons are ephemeral and show salinity swings from brackish to >300 g L^−1^ total dissolved solids within a single hydrological year [5]. Their chemistry is dominantly CaSO_4_–HCO_3_/CO_3_ rather than the NaCl signature typical of marine-derived brines, classifying them as athalassohaline systems [3]. Salineta and La Muerte represent two contrasting sites within this complex. Both occupy small (≤0.5 km^2^), shallow (<1 m) depressions floored by gypseous silts but differ subtly in hydrology. Salineta holds water for several months almost every year because its catchment receives occasional run-off from adjacent agricultural fields [6]. La Muerte, in contrast, is usually dry; episodic inundation depends largely on direct rainfall and a minor groundwater rise [7]. These hydrological differences determine the thickness, mineralogy, and redox zonation of the upper sediment centimeters, the micro-niche in which benthic microbes thrive. Evaporation-induced supersaturation supports repeated precipitation–dissolution cycles of gypsum, epsomite, and trona, creating sharp salinity and oxygen gradients over millimeter scales.

These extreme and fluctuating conditions exert strong selective pressures, fostering highly adapted and unique microbial communities [4]. Studies in the Monegros lagoons reveal a broad and often novel genetic diversity across Bacteria, Archaea, and microbial eukaryotes (protists) [3,4]. Ecological diversity for eukaryotes tends to be higher at intermediate salinities, while bacteria and archaea maintain diverse communities even at extreme salt concentrations [3,8]. The Monegros endorheic habitats serve as exceptional natural laboratories for studying microbial adaptation to poly-extreme conditions and ecosystem functioning in the face of environmental change. Their ecological value, coupled with their vulnerability to diffuse pollution from surrounding agricultural activities and the broader impacts of climate change, underscores the critical need for continued research to inform biomonitoring, conservation, and management strategies for these globally threatened ecosystems.

## 2. Materials and Methods

### 2.1. Sample Collection

**Figure 1:**
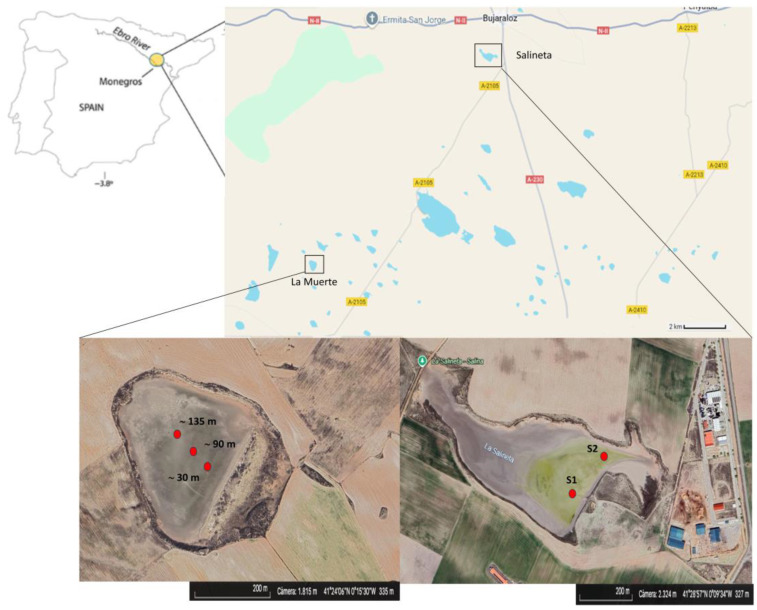
Geographical location of the Monegros desert endorheic area within the semi-arid Central Ebro Basin, NE Spain. Sampling locations at La Muerte lagoon and Salineta. Images captured by Google Earth.

The endorheic La Muerte lagoon is located at latitude 41.4013 and longitude −0.2614. Sediment cores with a diameter of 5 cm were collected to a depth of 1 cm. Immediately after collection, the samples were flash-frozen in liquid nitrogen, transported to the laboratory, and stored at −80 °C until further analysis. Sediment samples were obtained from several points approximately 30 m, 90 m, and 135 m from the lagoon’s border. Eighteen DNA extraction samples were grouped into six clusters, each composed of a mixture of three distinct samples spaced approximately 30 cm apart [7]. The groups were as follows: MS5 (January 2020, wet biofilm–sediment, 135 m), MS4 (September 2021, dry biofilm–sediment, 30 m), MS6 (July 2022, dry biofilm–sediment, 90 m), MS7 (April 2023, wet biofilm–sediment, 90 m), MS8 (June 2023, wet biofilm–sediment, 90 m), and MS9 (June 2023, wet biofilm–sediment, 135 m) (Figure 1).

The endorheic Salineta lagoon is located at latitude 41.4814 and longitude −0.1589. Raw sequencing data from Salineta lagoon were obtained from [6]. The selected sequences were derived from sediment cores of 7 cm diameter collected to a depth of 5 cm at two stations, S1 and S2, corresponding, respectively, to usually submerged soil and intermittently flooded soil. These stations were chosen as representative of water-related soil conditions in these ephemeral saline lakes [6]. Sediment samples were collected during winter (March 2019, “wet period”) and summer (September 2019, “dry period”).

From the available data, we selected two stations (S1, S2), one upper sediment layer (L1), and two seasons, each with three replicates: S1L1R1, S1L1R2, S1L1R3, S2L1R1, S2L1R2, S2L1R3, WS1L1R1, WS1L1R2, WS1L1R3, WS2L1R1, WS2L1R2, and WS2L1R3 (Bioproject PRJNA763109). These twelve samples were clustered into four groups—in analogy to the La Muerte lagoon samples—designated as S1, S2, WS1, and WS2 (Figure 1).

### 2.2. DNA Extraction and Amplicon Sequencing

DNA sample extraction from La Muerte was based on the protocol described by Berlanga et al. [7]. The DNA concentration was estimated using BioDrop µLite (Biotech, Madrid, Spain). Amplicon sequencing of the 16S rRNA gene (V3–V4 region) was performed on the Illumina MiSeq platform (MiSeq Nanorun 500 (2 × 250), San Diego, CA, USA). The primer pairs for region V3–V4 were 341F-CCTACGGGNGGCWGCAG and 805R-GACTACHVGGGTATCTAATCC [9].

### 2.3. Bioinformatic Analyses

Bioinformatic analysis was performed with sequences from La Muerte and selected sequences from Salineta. Raw sequence reads were processed, removing short read lengths, low quality scores, and chimeric reads, using USEARCH. Microbiota profiling was conducted with the 16S-based Microbial taxonomic profiling (MTP) platform of EzBio-Cloud Apps (ChunLab Inc., Seoul, Republic of Korea) (https://www.EZbiocloud.net, accessed on 19 September 2025). Taxonomic assignment of the reads was conducted with ChunLab’s 16S rRNA database (DB ver. PKSSU4.0) [10].

Bray–Curtis dissimilarity analysis was applied to measure beta diversity and to generate principal coordinate analysis plots and was conducted using the platform EzBioCloud. Differences were considered significant with *p* < 0.05. Biomarkers were determined using linear discriminant analysis (LDA) with effect size estimation (LefSe) [10].

The prediction of KEGG-based functional profiles from 16S-based taxonomic profiles was carried out within the MTP platform of EzBio-Cloud Apps (ChunLab Inc., Seoul, Republic of Korea) (https://www.EZbiocloud.net). KO genes refer to genes that have been assigned a KEGG Orthology (KO) identifier within the KEGG (Kyoto Encyclopedia of Genes and Genomes) database. The functional annotations should be considered preliminary and interpreted as putative activity potential. For the functionality study, we used data for S2 and WS2 (Salineta lagoon) with intermittently flooded soil, which may resemble conditions observed in La Muerte, which can also be wet and dry.

The method links 16S rRNA gene sequences with functional annotations from prokaryotic reference genomes through nearest-neighbor identification. Predicted functional abundances were normalized using 16S rRNA gene copy number estimates obtained from genome annotations to account for varying gene copy numbers across taxa. The individual KO genes detected were grouped into functional categories including metabolic pathways (carbon fixation, nitrogen cycling) and stress response systems following KEGG pathway classification. For example, we used relative abundance of KO key enzymes detected for 3-Hydroxypropionate (3-HP) Bi-Cycle (K14468), reverse TCA cycle (K15231), the Wood–Ljungdahl pathway (K14138), and the Calvin–Benson cycle (K01601). With respect to the nitrogen cycle, we included denitrification (K00374, K02568, K04561); nitrification (K10535, K10944, K10945, K10946), nitrogen fixation (K02586); dissimilatory sulfate reduction (K00394, K00395); osmotic stress (two-component system; Na+/H+ exchangers) (K07777, K07692, K07638, K07646, K07667, K07670, K07242, K02117, K16052); osmotic stress (compatible solute) (K02000, K02001, K02002, K02182, K03762, K10674, K00130, K03451, K05020); oxidative stress (K03782, K03386, K04761, K03781, K04564, K04565); starvation stress (K03087, K03048, K03049, K03050, K03088); and Polyhydroxybutirat (PHB) (K03821, K00023, K05973) [11]

Between-group differences in predicted functional capabilities were assessed using non-parametric statistical tests appropriate for compositional microbiome data. Mann–Whitney–Wilcoxon tests were applied to identify significantly different KO abundances between La Muerte and Salineta samples. Differences were considered significant with *p* < 0.05. Functional prediction represented a putative metabolic potential based on phylogenetic inference and should be interpreted cautiously. The accuracy of predictions depends on the availability of closely related reference genomes in the database. 

## 3. Results

### 3.1. Bacterial Diversity in La Muerte and Salineta Lagoons

**Figure 2:**
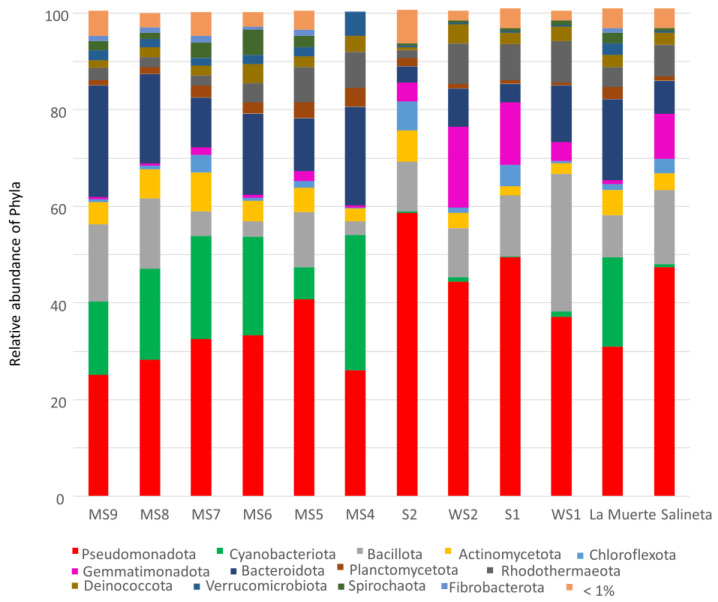
Proportions of bacterial phyla in biofilm–sediment samples from La Muerte (MS4, MS4, MS6, MS7, MS8, and MS9) and Salineta (S1, S2, WS1, and WS2) and the grouped samples from La Muerte and Salineta.

Sediment microbial communities in both La Muerte and Salineta lagoons exhibit clear taxonomic patterns. Pseudomonadota dominates all sediment samples across both lagoons. Across La Muerte sediments, Pseudomonadota averages 31.0% of sequences, peaking at 40.8% in the MS5 sample, while in Salineta sediments, Pseudomonadota averages 47.4% and reaches 58.6% in S2. Pseudomonadota is frequently a dominant phylum in saline and hypersaline environments, including artificial and natural saline water bodies, wetlands, and soils. The high prevalence of Pseudomonadota in the Monegros lagoons aligns with its well-documented metabolic adaptability and tolerance to osmotic stress [12]. Cyanobacteriota species comprise a significant fraction in La Muerte sediments (average 18.4%, up to 28.0% in MS4) but remain below 1% in Salineta samples, indicating stronger phototrophic sediment in biofilm–sediment in La Muerte [13]. Bacteroidota contributes markedly to the organic matter turnover (average 16.6%, up to 23.0% in MS9) compared with lower proportions in La Salineta (average 6.7%) (Figure 2). 

The La Muerte lagoon exhibits more stable and homogeneous bacterial communities, whereas Salineta stands out for its variability, particularly in the abundance of stress-adapted phyla including Gemmatimonadota and Deinococcota. Spirochaetota remains a consistently low but ecologically relevant component in both lagoons, aligned with its known sulfur-cycling functions in saline and micro-oxic environments [14,15]. Other phyla, including Actinomycetota and Planctomycetota, maintain a low but consistent presence (<8%).

**Figure 3:**
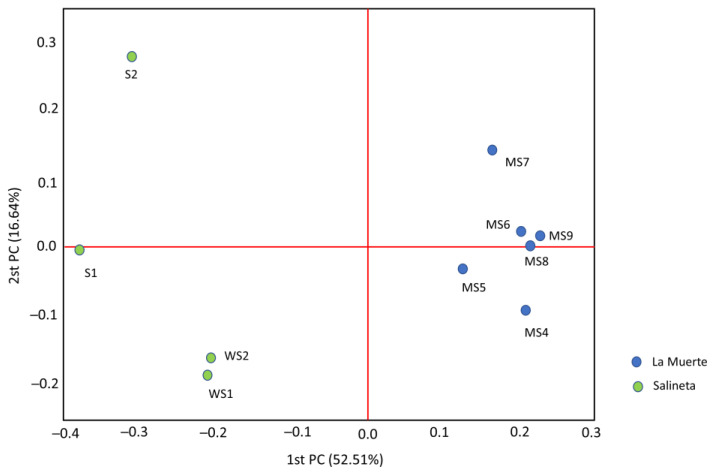
Principal coordinate analysis (PCoA) of the bacteria community distribution in La Muerte samples (blue circles) and Salineta samples (green circles). Bray–Curtis dissimilarity was employed to measure the differences in community composition. Each point represents a distinct sample, with the spatial arrangement reflecting the dissimilarity in microbial composition—closer points indicate more similar communities (Bray–Curtis dissimilarity *p* < 0.05).

The principal coordinate analysis (PCoA) compared the microbial beta-diversity between La Muerte and Salineta lagoons in the Monegros desert, revealing strong community differentiation (Figure 3). Microbial communities in La Muerte and Salineta lagoons are not only distinct from each other but also vary in their internal diversity. The tight grouping of La Muerte samples points to stable conditions or a strong selection pressure resulting in a uniform community. In contrast, greater spread among Salineta samples might reflect more dynamic conditions, environmental heterogeneity, or episodic ecological disturbances. These habitat-specific microbiota suggested that environmental factors unique to each lagoon strongly influence the composition and structure of its microbiota.

La Muerte’s homogeneity likely stems from its stable substrate, limited vertical structuring, and a core microbial community well adapted to oscillating aridity and flooding. La Muerte may be more resistant to short-term disturbances. Salineta may maintain flexible taxa to occupy a changing habitat with potential for rapid response (or abrupt shifts) under external pressures such as pollution or hydrological alteration.

LEfSe (linear discriminant analysis effect size) identifies taxa that are statistically and biologically relevant different between groups—in this case, between the La Muerte and Salineta lagoons. Table S1 (Supplementary Materials) lists bacterial families with their LDA effect sizes, *p*-values, FDR-corrected *p*-values, and relative abundances in both lagoons; 25 families were enriched in La Muerte vs. 13 in Salineta, based on a higher mean abundance per family. La Muerte displays significant enrichment of Cyanobacteriota, emphasizing the importance of photosynthetic bacteria in this lagoon. Phyla such as Bacteroidota and Planctomycetota highlight differences in organic matter decomposition and nutrient cycling functions. On the other hand, in Salineta, enriched families include Arenicellaceae, Thermoleophilaceae, Ectothiorhodospiraceae, Desulfovibrionaceae, Moritellaceae, Sphaerobacteraceae, suggesting salt-, sulfur- and anaerobe-adapted taxa, and potential hydrocarbon or refractory carbon degraders under saline conditions. The presence of high abundances of Pseudomonadota-associated taxa (e.g., Alphaproteobacteria) suggests active sulfur and nitrogen cycling, often linked with microoxic or highly saline niches.

### 3.2. Putative Functionality in La Muerte and Salineta Lagoons

The carbohydrate index profiles indicate contrasting organic matter quality and polymer composition between lagoons, with La Muerte showing relatively higher signals tied to glucose over phenolic background and a greater contribution of xylose relative to glucose, consistent with distinct monosaccharide balance or hemicellulosic inputs. Salineta, in turn, shows a relatively higher glucose-to-phosphate balance and a stronger combined glucose + xylose signal relative to cellulose, which is compatible with a comparatively more polymer-rich or cellulose-linked carbohydrate pool and a different phosphorylation/energy status of dissolved or particulate carbohydrates (Table 1) [16]. Together, these indices support a scenario in which carbon sources and polymerization states differ markedly between lagoons, with implications for decomposition rates and microbial substrate preferences.

**Figure 4:**
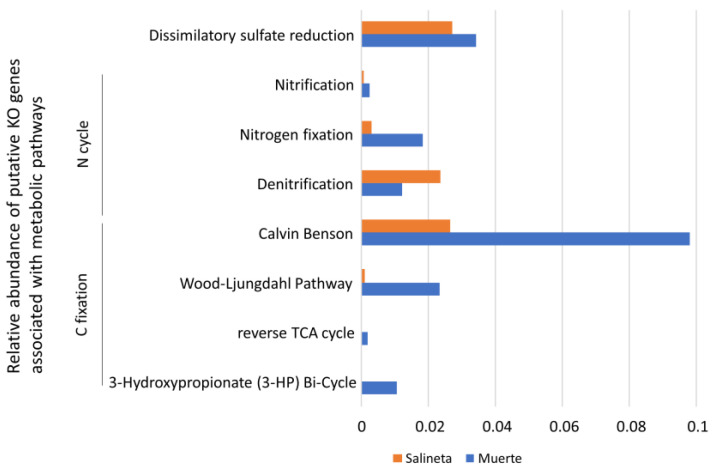
Bar graph illustrating the relative abundance of putative KEGG Orthology (KO) genes associated with metabolic pathways involved in carbon fixation, nitrogen cycling, and dissimilatory sulfate reduction across grouped samples from La Muerte (MS4–MS9) and Salineta (S2 and WS2). All stress-related KO genes had significant differences (Mann–Whitney–Wilcoxon test, *p* < 0.05).

The analysis of several putative metabolic pathways (Figure 4) revealed that La Muerte exhibited a more diverse repertoire of autotrophic carbon fixation capabilities, with signatures consistent with multiple CO_2_-fixing pathways (Calvin–Benson cycle, Wood–Ljungdahl pathway, 3-hydorxypropionate bi-cycle, and reverse TCA), accompanied by enhanced potential for nitrification and nitrogen fixation compared to Salineta. In contrast, Salineta showed a more pronounced denitrification signature, indicating a greater prevalence of respiratory nitrate reduction under the site-specific redox conditions. These metabolic profiles suggested that la Muerte communities were more dependent on autotropic primary production and endogenous nitrogen cycling, while Salineta communities were predominantly functioning under anoxic conditions.

**Figure 5:**
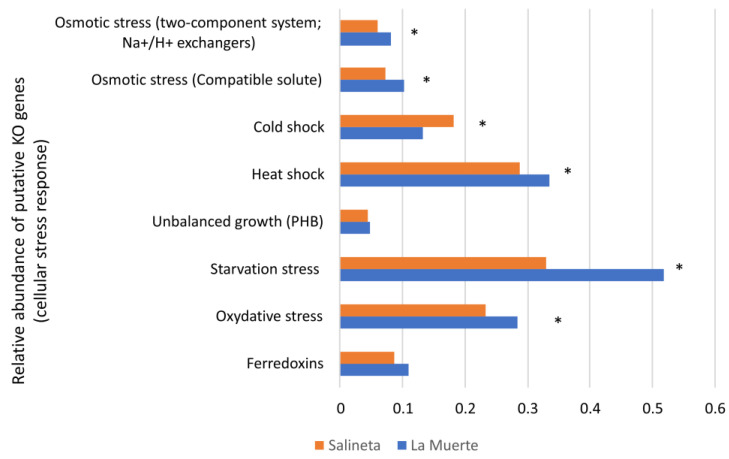
Putative OK genes related to several stress categories. Bar graph illustrating the relative abundance of putative KEGG Orthology (KO) genes involved in various cellular stress responses across grouped samples from La Muerte (MS4–MS9) and Salineta (S2 and WS2). Asterisk indicates significant differences (Mann–Whitney–Wilcoxon test, *p* < 0.05).

Because each stress category aggregates a different number of putative KOs, only within-category comparisons are appropriate (Figure 5). Most stress categories show higher values at La Muerte than at Salineta—e.g., starvation (≈57% higher), osmotic adaptation via compatible solutes (≈44% higher), and Na^+^/H^+^–linked osmotic systems (≈38% higher)—with the notable exception of cold shock, which is higher at Salineta (0.181 vs. 0.133; ≈27% higher), suggesting site-specific differences in temperature variability or cold exposure. Monegros saline wetlands are fed by distinct aquifers and vary in basin morphology and connectivity, producing site-to-site differences in hydroperiod and salinity that can elevate stress exposure where groundwater support is weaker or evaporation dominates, as expected for some ephemeral basins like La Muerte [17].

## 4. Discussion

### 4.1. Bacterial Diversity and Putative Functionality in La Muerte and Salineta Lagoons

The comparative analysis of microbial communities between the La Muerte and Salineta sites reveals profound differences in taxonomic composition, metabolic capabilities, and stress response mechanisms that reflect distinct environmental pressures and adaptive strategies. These findings contribute to our understanding of how microbial ecosystems respond to varying environmental conditions and provide insights into the fundamental principles governing microbial community assembly and function in contrasting habitats.

The LEfSe analysis reveals pronounced, ecologically relevant differences in the dominant bacterial families between the La Muerte and Salineta lagoons (Table S1, Supplementary Materials). These differences likely reflect adaptation to local environmental conditions and have significant implications for ecosystem functioning, biogeochemical cycling, and the resilience of these unique saline habitats. The identified biomarkers offer valuable tools for monitoring and managing the ecological health and stability of these saline lagoon ecosystems. The dominance of cyanobacterial taxa in La Muerte is consistent with findings from other extreme environments where these organisms serve as primary producers and nitrogen fixers under stressful conditions [18]. The enrichment of Pseudomonadota families in Salineta indicates a different ecological strategy, potentially related to heterotrophic metabolism and organic matter processing. The presence of sulfur-metabolizing families like Desulfuromonadaceae and Desulfovibrionaceae with higher relative abundance in Salineta suggests an active sulfur cycling, which is characteristic of marine and hypersaline environments where sulfate reduction plays a crucial role in biogeochemical processes [19,20].

The metabolic pathway analysis revealed differences in energy acquisition and nutrient cycling strategies between the two sites (Figure 3). La Muerte showed significantly higher Calvin–Benson cycle putative activity (0.098 vs. 0.027), indicating enhanced autotrophic carbon fixation capacity. This elevated carbon fixation potential correlates with the abundance of cyanobacterial taxa and suggests that La Muerte functions as an autotroph-dominated ecosystem [21]. Conversely, Salineta exhibited higher putative denitrification activity (0.024 vs. 0.012), suggesting more active nitrogen cycling under potentially oxygen-limited or nitrate-rich conditions. This pattern is consistent with findings from marine environments where denitrification becomes prominent under specific redox conditions [22]. Complementary redox measurements (not included in this dataset) indicate that La Muerte typically presents moderately positive values (approximately +337 to +125 mV, depending on inundation), whereas Salineta frequently develops much lower redox potentials, occasionally reaching strongly reducing conditions (−178 to −446 mV in interstitial waters during summer brine phases). These patterns suggest that Salineta can sustain anoxic microenvironments conducive to denitrification, while La Muerte generally dries too rapidly for such conditions to stabilize. We therefore believe that the functional inferences derived from metagenomic signatures are consistent with the environmental context observed in both lagoons. The putative dissimilatory sulfate reduction showed relatively similar levels between sites, but slightly higher activity in La Muerte (0.034 vs. 0.021), indicating active sulfur cycling across both environments.

The most ecologically relevant stress categories for the endorheic saline lagoons of Monegros are osmotic stress and ion homeostasis, temperature stress (both heat and cold shocks), oxidative/UV stress, nutrient/energy limitation, and redox balancing linked to ferredoxins and storage polymers like polyhydroxybutyrate (PHB). PHB accumulates under unbalanced growth (e.g., nutrient limitation with excess carbon or elevated salinity), serving as a carbon/electron sink that can buffer redox stress and energy imbalances [23]. These priorities reflect the lagoons’ shallow, highly evaporative, and seasonally variable hydrology, which imposes large salinity and temperature swings alongside strong irradiance and an intermittent nutrient supply [3].

Osmotic stress is primarily observed in athalassohaline settings, where fluctuating and sometimes high salinities select for compatible-solute strategies and Na^+^/H^+^ exchange to maintain water balance and cytoplasmic pH, a hallmark of halophile ecology also evident in Monegros biofilm–sediment communities. Redox and depth-related gradients at Salineta further indicate rapid environmental change near the surface, reinforcing the need for robust osmoadaptation and ion homeostasis under dynamic salinity and oxygen conditions [6,7,24].

Endorheic saline lagoons often experience nutrient limitation alongside episodic inputs; in Monegros, diffuse agricultural and livestock pollution can superimpose on oligotrophic baselines, altering microbial redox processes and stress phenotypes. Such interacting drivers plausibly underpin stronger starvation and oxidative stress in the La Muerte lagoon vs. Salineta, reflecting differences in organic matter supply, electron acceptor availability, and contaminant pressure along the landscape [6,17].

La Muerte showed higher within-category stress signals than Salineta; it is possible that its ephemeral, shallow, and highly evaporative setting may impose stronger osmotic, thermal, oxidative, and nutrient limitation pressures, whereas Salineta’s vertically structured biotope provides more environmental buffering that could reduce the overall stress intensity [6,7,25].

These contrasting strategies reflect fundamental ecological principles governing microbial community assembly under different environmental pressures. 

### 4.2. Perspectives, Implications, and Future Research Directions

This comparative analysis contributes to our understanding of microbial ecosystem differentiation and offers insights into how environmental conditions influence community structure and function. The observed differences between La Muerte and Salineta indicated that specific environmental situations may shape distinct ecosystem functioning modes, with potential implications for biogeochemical cycling and community dynamics. The observed relation between stress response capabilities and environmental conditions may indicate that microbial communities could serve as bioindicators of ecosystem health and environmental variation, although further validation across different systems would be necessary to establish this relationship more definitively.

In future research, longitudinal studies would be valuable for examining how these communities respond to temporal variations and environmental change over extended periods that could provide insights into the stability and resilience of these ecosystem types under varying conditions. Given projected changes in temperature, salinity, and extreme weather patterns, examining the resilience and adaptive capacity of microbial systems could inform ecosystem management approaches. However, the complexity of environmental interactions and the site-specific nature of microbial communities’ extrapolation should be approached with appropriate caution.

## Figures and Tables

**Table 1:** Carbohydrate index.

	GLU:PHEN	GLU:PHOS	XYL:GLU	(GLU + XYL): CEL
La Muerte	4.8	1.05:1	0.1588	5.77
Salineta	1.02	2.2:1	0.0134	53.3

Beta-Glucosidase-Phenol oxidase index (GLU:PHEN), referred to as the recalcitrant index. Beta-Glucosidase-Phosphatase (GLU:PHOS); 1:1 ratios indicate an equilibrium between the elemental composition of available organic matter. It can be used to detect P limitation. Beta-Xylosidase:Beta-glucosidase (XYL:GLU) indicates the origin of polysaccharides; high values may indicate greater use of allochthonous plant material. (β-Glucosidase + β-xylosidase): cellobiohydrolase. (GLU + XYL): CEL shows the complexity of the available polysaccharides; higher values indicate higher capacity for decomposition of simple polysaccharides [16].

## Data Availability

The data presented in this study are openly available in NCBI database at https://www.ncbi.nlm.nih.gov/sra/, reference number [PRJNA962491].

## Supplementary Materials

The following supporting information can be downloaded at https://www.mdpi.com/article/10.3390/microorganisms13102224/s1, Table S1: Differential abundance analysis of bacterial families between La Muerte and Salineta lagoons using LEfSe.
