# Supplementary material for: Comparative Analysis of Bacterial Diversity and Functional Potential in Two Athalassohaline Lagoons in the Monegros Desert (NE Spain)
Source: Microorganisms. 2025 Sep 23;13(10):2224. doi: 10.3390/microorganisms13102224 (PMC12566095; doi:10.3390/microorganisms13102224)
Supplement: Supplementary file 1 [file microorganisms-13-02224-s001.zip › microorganisms-3878618-supplementary.pdf]

Taxon name	Taxonomy	LDA effect size	p-value	p-value (FDR)	La Muerte	Salineta
Chelatococcaceae	Bacteria: Proteobacteria: Alphaproteobacteria: Rhizobiales	4,51309	0,01052	0,17547	6,70282	0,27212
Prochlorotrichaceae	Bacteria: Cyanobacteria: Chroobacteria: Oscillatoriales	4,44356	0,01028	0,17547	5,41973	0,01271
Microcystaceae	Bacteria: Cyanobacteria: Chroobacteria: Chroococcales	4,05392	0,0096	0,17547	1,8998	0,00279
Arenicellaceae	Bacteria: Proteobacteria: Gammaproteobacteria: Arenicellales	3,83457	0,00635	0,17547	0,00084	1,68192
Pseudanabaenaceae	Bacteria: Cyanobacteria: Chroobacteria: Oscillatoriales	3,8069	0,0083	0,17547	1,40941	0
Catalimonadaceae	Bacteria: Bacteroidetes: Cytophagia: Cytophagales	3,77038	0,01028	0,17547	1,30088	0,00603
Rubricoccaceae	Bacteria: Rhodothermaeota: Rhodothermia: Rhodothermales	3,75035	0,01052	0,17547	1,29326	0,16066
Thermoleophilaceae	Bacteria: Actinobacteria: Thermoleophilia: Thermoleophilales	3,67068	0,0083	0,17547	0,00157	0,99566
Puniceicoccaceae	Bacteria: Verrucomicrobia: Opitutae: Puniceicoccales	3,67047	0,01052	0,17547	1,17684	0,14858
Ectothiorhodospiraceae	Bacteria: Proteobacteria: Gammaproteobacteria: Chromatiales	3,55156	0,03301	0,25591	0,19355	0,92007
Desulfuromonadaceae	Bacteria: Proteobacteria: Deltaproteobacteria: Desulfuromonadales	3,49942	0,01052	0,17547	0,04327	0,65805
Desulfovibrionaceae	Bacteria: Proteobacteria: Deltaproteobacteria: Desulfovibrionales	3,42212	0,03248	0,25591	0,5105	0,00805
Bacillaceae	Bacteria: Firmicutes: Bacilli: Bacillales	3,36307	0,01902	0,19358	0,27473	0,73939
Bradyrhizobiaceae	Bacteria: Proteobacteria: Alphaproteobacteria: Rhizobiales	3,33878	0,00635	0,17547	0,00106	0,48014
Egibacteraceae	Bacteria: Actinobacteria: Nitriliruptoria: Egibacterales	3,32639	0,01028	0,17547	0,43581	0,02063
Parvularculaceae	Bacteria: Proteobacteria: Alphaproteobacteria: Parvularculales	3,29062	0,0083	0,17547	0,40706	0
Caldilineaceae	Bacteria: Chloroflexi: Caldilineae: Caldilineales	3,1932	0,01052	0,17547	0,33154	0,03036
Hyphomonadaceae	Bacteria: Proteobacteria: Alphaproteobacteria: Rhodobacterales	3,18119	0,0083	0,17547	0,33399	0
Chthoniobacteraceae	Bacteria: Verrucomicrobia: Spartobacteria: Chthoniobacterales	3,1011	0,0083	0,17547	0,25366	0
Gottschalkiaceae	Bacteria: Firmicutes: Clostridia: Clostridiales	3,08204	0,04119	0,25591	0,00042	0,1798
Oxalobacteraceae	Bacteria: Proteobacteria: Betaproteobacteria: Burkholderiales	3,05637	0,01052	0,17547	0,00786	0,2604
Haliscomenobacteraceae	Bacteria: Bacteroidetes: Sphingobacteriia: Saprospirales	2,99987	0,01028	0,17547	0,21626	0,01271
Methylobacteriaceae	Bacteria: Proteobacteria: Alphaproteobacteria: Rhizobiales	2,99493	0,01028	0,17547	0,00198	0,22775
Isosphaeraceae	Bacteria: Planctomycetes: Planctomycetia: Planctomycetales	2,92049	0,01052	0,17547	0,19096	0,0311
Moritellaceae	Bacteria: Proteobacteria: Gammaproteobacteria: Alteromonadales	2,85529	0,01824	0,19358	0	0,11195
Nannocystaceae	Bacteria: Proteobacteria: Deltaproteobacteria: Myxococcales	2,78169	0,01028	0,17547	0,13092	0,00187
Woeseiaceae	Bacteria: Proteobacteria: Gammaproteobacteria: Chromatiales	2,77567	0,01052	0,17547	0,00834	0,13825
Salinivirgaceae	Bacteria: Bacteroidetes: Bacteroidia: Marinilabiliales	2,72272	0,03664	0,25591	0,0943	0,00119
Sphaerobacteraceae	Bacteria: Chloroflexi: Thermomicrobia: Sphaerobacterales	2,69441	0,01824	0,19358	0	0,08157
Oscillatoriaceae	Bacteria: Cyanobacteria: Chroobacteria: Oscillatoriales	2,685	0,0083	0,17547	0,1017	0
Alcanivoracaceae	Bacteria: Proteobacteria: Gammaproteobacteria: Oceanospirillales	2,61567	0,03301	0,25591	0,11848	0,01022
Bdellovibrionaceae	Bacteria: Proteobacteria: Oligoflexia: Bdellovibrionales	2,5585	0,0083	0,17547	0,07815	0
Ignavibacteriaceae	Bacteria: Chlorobi: Ignavibacteriae: Ignavibacteriales	2,35607	0,03664	0,25591	0,04511	0,00213
Alteromonadaceae	Bacteria: Proteobacteria: Gammaproteobacteria: Alteromonadales	2,31322	0,03248	0,25591	0,05524	0,00434
Leptospiraceae	Bacteria: Spirochaetes: Spirochaetia: Leptospirales	2,24461	0,02295	0,19663	0,03476	0
Thioprofundaceae	Bacteria: Proteobacteria: Gammaproteobacteria: Chromatiales	2,20192	0,0096	0,17547	0,034	0,00069
Oligoflexaceae	Bacteria: Proteobacteria: Oligoflexia: Oligoflexales	2,19421	0,03248	0,25591	0,04	0,0059
Prevotellaceae	Bacteria: Bacteroidetes: Bacteroidia: Bacteroidales	2,09119	0,01902	0,19358	0,00642	0,03003
